# A practical dynamic nomogram model for predicting bone metastasis in patients with thyroid cancer

**DOI:** 10.3389/fendo.2023.1142796

**Published:** 2023-03-06

**Authors:** Wen-Cai Liu, Meng-Pan Li, Wen-Yuan Hong, Yan-Xin Zhong, Bo-Lin Sun, Shan-Hu Huang, Zhi-Li Liu, Jia-Ming Liu

**Affiliations:** ^1^ Department of Orthopaedic Surgery, The First Affiliated Hospital of Nanchang University, Nanchang, China; ^2^ The First Clinical Medical College of Nanchang University, Nanchang, China; ^3^ Department of Orthopaedics, Shanghai Jiao Tong University Affiliated Sixth People’s Hospital, Shanghai, China; ^4^ Institute of Spine and Spinal Cord, Nanchang University, Nanchang, China

**Keywords:** nomogram, prediction, bone metastasis, thyroid cancer, risk

## Abstract

**Purpose:**

The aim of this study was to established a dynamic nomogram for assessing the risk of bone metastasis in patients with thyroid cancer (TC) and assist physicians to make accurate clinical decisions.

**Methods:**

The clinical data of patients with TC admitted to the First Affiliated hospital of Nanchang University from January 2006 to November 2016 were included in this study. Demographic and clinicopathological parameters of all patients at primary diagnosis were analyzed. Univariate and multivariate logistic regression analysis was applied to build a predictive model incorporating parameters. The discrimination, calibration, and clinical usefulness of the nomogram were evaluated using the C-index, ROC curve, calibration plot, and decision curve analysis. Internal validation was evaluated using the bootstrapping method.

**Results:**

A total of 565 patients were enrolled in this study, of whom 25 (4.21%) developed bone metastases. Based on logistic regression analysis, age (OR=1.040, *P*=0.019), hemoglobin (HB) (OR=0.947, *P*<0.001) and alkaline phosphatase (ALP) (OR=1.006, *P*=0.002) levels were used to construct the nomogram. The model exhibited good discrimination, with a C-index of 0.825 and good calibration. A C-index value of 0.815 was achieved on interval validation analysis. Decision curve analysis showed that the nomogram was clinically useful when intervention was decided at a bone metastases possibility threshold of 1%.

**Conclusions:**

This dynamic nomogram, with relatively good accuracy, incorporating age, HB, and ALP, could be conveniently used to facilitate the prediction of bone metastasis risk in patients with TC.

## Introduction

Thyroid cancer (TC) is an uncommon endocrine cancer that accounts for approximately 1% of all new malignancies, roughly 0 - 5% of cancers in men and 1 - 5% of cancers in women (1–3). However, the incidence of thyroid cancer has been increasing for more decades (4). In the United States, the incidence of thyroid cancer tripled from 4.5 per 100,000 population in 1974 to 14.4 in 2013 (5). Differentiated thyroid cancer (DTC) of low malignancy accounted for the highest proportion of thyroid cancer (90%), including papillary carcinoma (70-75%) and follicular carcinoma (15-20%) (6). Undifferentiated carcinomas, which are anaplastic malignancies, accounts for less than 5% (6, 7). Therefore, the prognosis of patients with thyroid cancer is generally good, with a 10-year survival rate of 80-95% (8). Distant metastasis is an important risk factor for patients with thyroid cancer. Compared with simple DTC patients, the 10-year survival rate of patients with distant metastasis is decreased by about 50% (3, 8). Bone is the third most common metastasis site in patients with TC, occurring in 2-13% of DTC patients (9–11) Compared with other distant metastases, bone metastases cause bone pain, pathological fractures and spinal cord compression, which significantly impaired their quality of life (12). Early diagnosis and intervention in such patients was important role for increasing patient survival rates (13, 14).

Bone scintigraphy and other nuclear studies, such as FDG-PET and SPECT, have high sensitivity and specificity for the early detection of bone metastases (15). However, their use is often limited due to high cost and radiation damage to patients (16). Thus, it is of great significance to develop a simple and feasible new method for early prediction of thyroid cancer bone metastasis. Nomograms have proven useful as models for predicting the occurrence of clinical events, and can allow visualization of incidence (17). In this study, we aimed to developed a valid nomogram model for assessing the risk of bone metastases in patients with TC to assist physicians in making accurate clinical decision.

## Materials and methods

### Patient information

**Figure 1:**
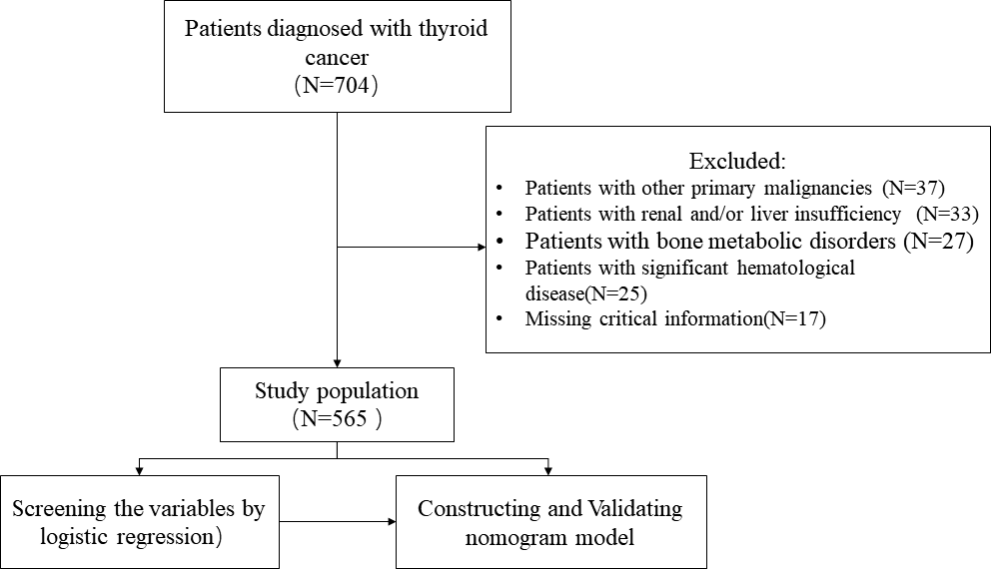
The study flow chart of case screening.

This study was approved by the Ethics Committee of the First Affiliated Hospital of Nanchang University, and all participants signed written informed consent form. From January 2006 to November 2016, patients newly diagnosed with TC in our hospital were included in this study. All diagnoses were confirmed by needle biopsy or open surgical biopsy. The exclusion criteria were as follows: (1) Patients with other primary malignancies; (2) Patients with renal and/or liver insufficiency; (3) Patients with bone metabolic disorders; (4) Patients with significant hematological disease; (5) Missing critical information. The detailed screening process is shown in **Figure 1**.

Bone scintigraphy was used to identify possible bone metastases in patients. If necessary, magnetic resonance imaging and local computed tomography were conducted to confirm possible diagnoses of bone metastases.

### Data collection

Demographic and clinicopathological parameters of all patients at primary diagnosis (before receiving clinical treatment) were collected, including age, serum concentrations of calcium, hemoglobin (HB), free triiodothyronine, free thyroxine 4, thyroid stimulating hormone, alkaline phosphatase (ALP), and common tumor markers (carcinoembryonic antigen, alpha fetoprotein, cancer antigen-125 (CA125), CA153, and CA199). Correlations between clinicopathological parameters and bone metastases were analyzed in patients with TC.

### Statistical analysis

All statistical analyses were performed using SPSS (version 26) and R (version 3.6.3) software. Qualitative variables were analyzed by Chi-square test, and quantitative variables were analyzed by Student’s t-test. Univariate analyses were initially used to identify variables that may affect bone metastases, and correlated variables (*P*<0.05) were included in multivariate logistic regression analysis to identify independent predictors of bone metastasis of thyroid cancer. Then, the selected independent risk factors were used to construct a dynamic nomogram for predicting bone metastases. C-index, ROC curve, calibration plot, and decision curve analysis were used to evaluate the performance and clinical usefulness of the model. The consistency index, Harrell’s C-index, was used to evaluate the predictive performance of the nomogram. And a ROC curve was established to compare the performance of nomogram model with independent predictors. Further, the nomogram was subjected to bootstrapping validation with 1,000 replications, to calculate an adjusted C-index and a calibration curve was used to judge predictive consistency. Decision curve analysis was conducted to determine the clinical usefulness of the nomogram by quantifying the net benefits at different probability thresholds.

## Results

### Demographic and pathological characteristics

A total of 565 patients with TC were enrolled in this study, of whom 25 (4.21%) had bone metastases and 540 (95.79%) had no bone metastases at initial diagnosis. Patient clinical characteristics are detailed in **Table 1**. Age, ALP and HB were significantly different between the bone metastasis group and the non-bone metastasis group (*P*<0.05). The average age, ALP and HB in the bone metastasis group were 53 years, 119.68U/L and 101.23g/L, respectively. The average age, ALP and HB in the non-bone metastasis group were 43.37 years, 68.83U/L and 123.07g/L, respectively.

**Table 1:** Comparison of clinical characteristics between bone metastasis and non-bone metastasis groups.

Characteristics	BM	NBM	*P* value
**Age(year)**	53.00 ± 14.12	43.37 ± 14.78	0.003
**Gender (%)**			0.763
**Male**	6(24.0)	137(25.36)	
**Female**	19(76.0)	403(74.63)	
**HB(g/L)**	101.23 ± 24.75	123.07 ± 17.16	<0.001
**ALP (U/L)**	119.68 ± 100.64	68.83 ± 49.30	0.028
**FT3**	2.78± 1.33	3.32± 5.63	0.691
**FT4**	1.11 ± 0.45	2.25 ± 2.70	0.083
**TSH**	15.31 ± 29.87	7.30 ± 19.28	0.288
**Ca(mmol/L)**	2.19± 0.43	2.22 ± 0.28	0.558
**CA125 (u/ml)**	65.04 ± 13.46	38.66 ± 25.09	0.658
**CA153 (u/ml)**	11.67 ± 5.33	19.71 ± 29.48	0.515
**CA199 (u/ml)**	21.57 ± 23.48	30.09 ± 20.11	0.803
**CA724 (u/ml)**	0.75 ± 0.02	2.72± 1.14	0.191
**CEA (ng/ml)**	55.31 ± 13.72	32.43 ± 21.89	0.412
**Cyfra21-f**	7.96 ± 4.04	2.78+ ± 1.99	0.310
**NSE**	18.60 ± 3.20	23.13 ± 9.44	0.839
**Histopathology**			0.661
Micro papillary carcinoma	17(68.0)	406(75.3)	
Eosinophilic follicular carcinoma	1(4.0)	11(2.0)	
Medullary carcinoma	4(16.0)	17(3.1)	
Undifferentiated carcinoma	0	13(2.4)	
Follicular papillary carcinoma	1(4.0)	68(12.6)	
Adenocarcinoma	2(8.0)	25(4.6)	
**Location of primary tumor**			0.945
Left thyroid cancer	8(32.0)	195(36.1)	
Right thyroid cancer	13(52.0)	246(45.6)	
Thyroid cancer of isthmus	0	5(0.9)	
Bilateral thyroid cancer	4(16.0)	94(17.4)	

BM, Bone metastasis; NBM, No bone metastasis; HB, hemoglobin; ALP, alkaline phosphate.

### Parameter selection

Logistic regression analysis was used to screen parameters. Univariate logistic regression analysis showed that age, ALP and HB were significantly correlated with bone metastasis of TC (p<0.05). Then the three related variables were included in the multivariate logistic regression analysis, and the results showed that age, ALP and HB were independent risk factors of bone metastasis of TC (P<0.05), so these three variables were used to construct the nomogram model (**Table 2**).

**Table 2:** Univariate analysis and multivariate analysis for prediction factors of bone metastasis in patients with thyroid cancer.

Factors	Univariate analysis			Multivariate analysis		
	β	OR (95%CI)	*P* value	β	OR (95%CI)	*P* value
Age	0.045	1.046 (1.015-1.078)	0.004*	0.039	1.040 (1.006-1.074)	0.019*
Gender	-0.157	0.855 (0.301-2.364)	0.762			
HB(g/L)	-0.057	0.944 (0.924-0.965)	<0.001*	-0.06	0.947 (0.926-0.968)	<0.001*
ALP (U/L)	0.006	1.007 (1.002-1.011)	0.007*	0.006	1.006 (1.002-1.010)	0.002*
FT3	-0.140	0.870 (0.561-1.347)	0.532			
FT4	-0.959	0.383 (0.120-1.222)	0.105			
TSH	0.013	1.013 (0.997-1.030)	0.120			
Ca(mmol/L)	-0.412	0.663 (0.168-2.616)	0.557			
CA125 (u/ml)	0.001	1.001 (0.997-1.005)	0.663			
CA153 (u/ml)	-0.023	0.977 (0.907-1.052)	0.542			
CA199 (u/ml)	-0.003	0.997 (0.980-1.015)	0.766			
CEA (ng/ml)	0.007	1.007 (0.999-1.016)	0.089			
Cyfra21-f	0.873	2.395 (0.606-9.467)	0.213			
NSE	-0.009	0.991 (0.918-1.070)	0.822			
Histopathology	0.056	1.058 (0.823-1.359)	0.661			
Location of primary tumor	-0.014	0.986 (0.658-1.477)	0.945			

β, coefficient of regression; OR, odds ratio; CI, confidence interval. * means that the P value is less than 0.05 (P<0.05).

### A dynamic nomogram model for predicting bone metastases

**Figure 2:**
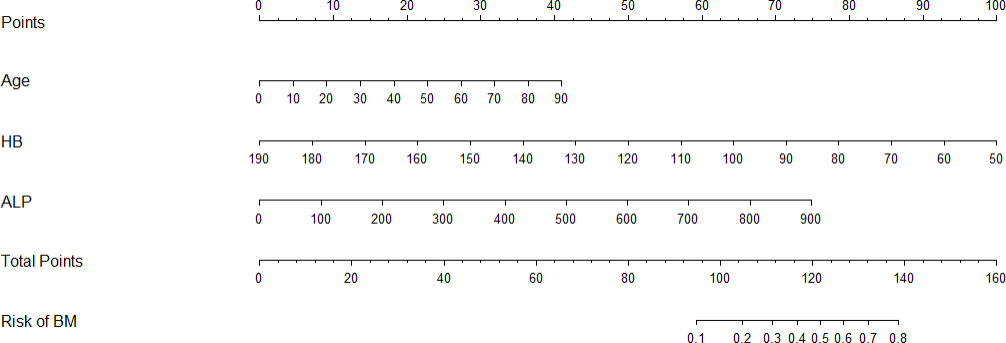
Nomogram for prediction of bone metastases. Bone metastases prediction nomogram, developed based on patient age, HB, and ALP levels.

**Figure 3:**
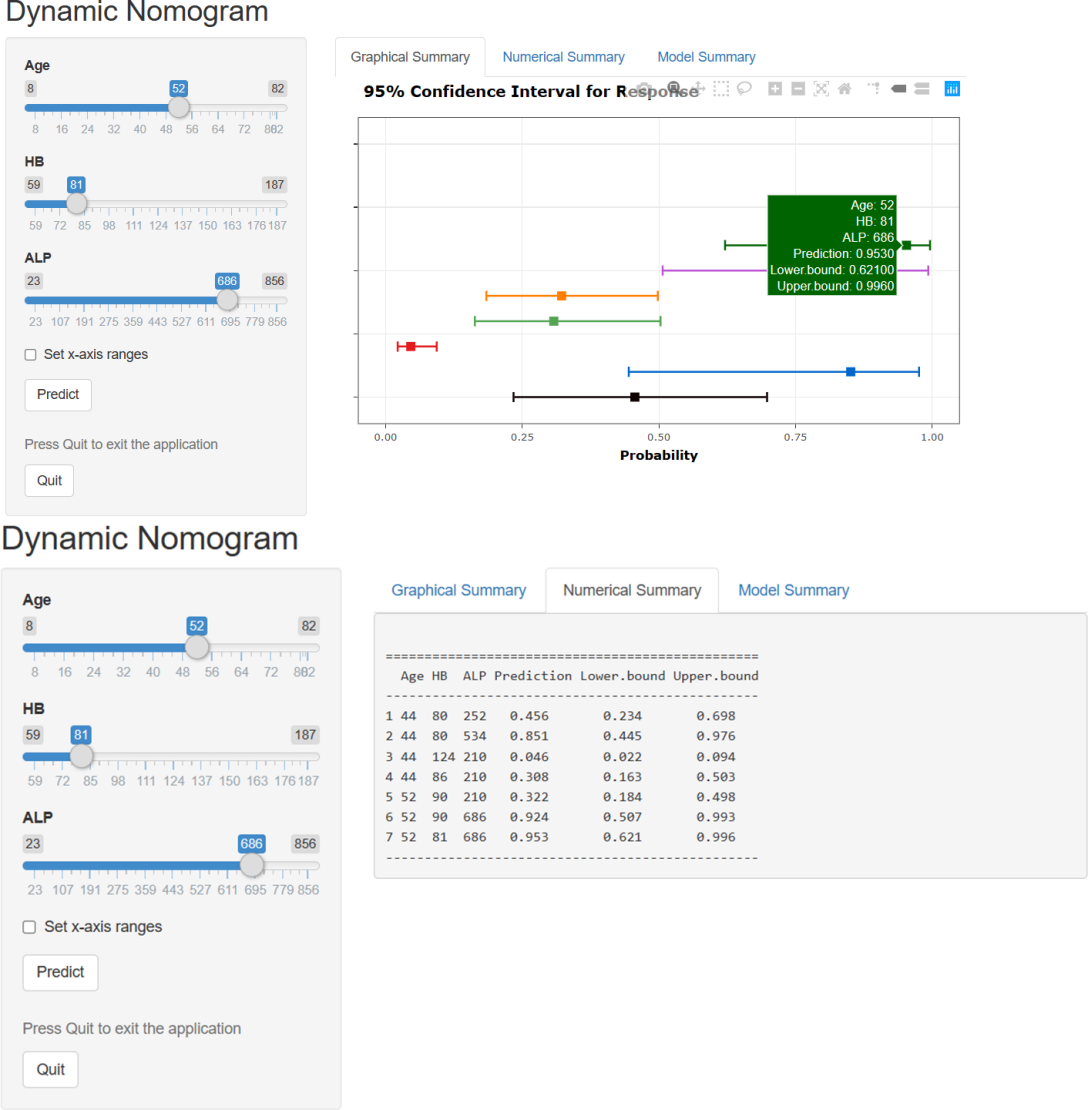
The dynamic nomogram for prediction of bone metastases. The plot displays probability (with 95% confidence interval) of bone metastases for patients with TC. The actual explanatory values and their corresponding predictions are given in the “Numerical Summary” tab.

The results of logistic regression analysis including age, ALP, and HB are presented in **Table 2**. A model that incorporated the above independent predictors was developed and presented as a nomogram (**Figure 2**). For patients with TC, the total points calculated using the nomogram could be visually converted to the risk of bone metastases. However, the nomogram can become more cumbersome to read and use when there are higher order interaction and smoothers items in the model. To better address this issue, we established a dynamic nomogram based on shiny to simplify the operation of users (**Figure 3**) **(** 18). The dynamic nomogram can be used to predict the probability of bone metastasis (and corresponding 95% Confidence interval) for any combination of predictor values. The shiny tabs display the predicted values graphically and numerically, and the model summary presents the underlying information of the model. Everyone can simply use the dynamic nomogram by clicking on the hyperlink (https://liuwencai.shinyapps.io/thyroid/).

### Apparent performance of the nomogram

**Figure 4:**
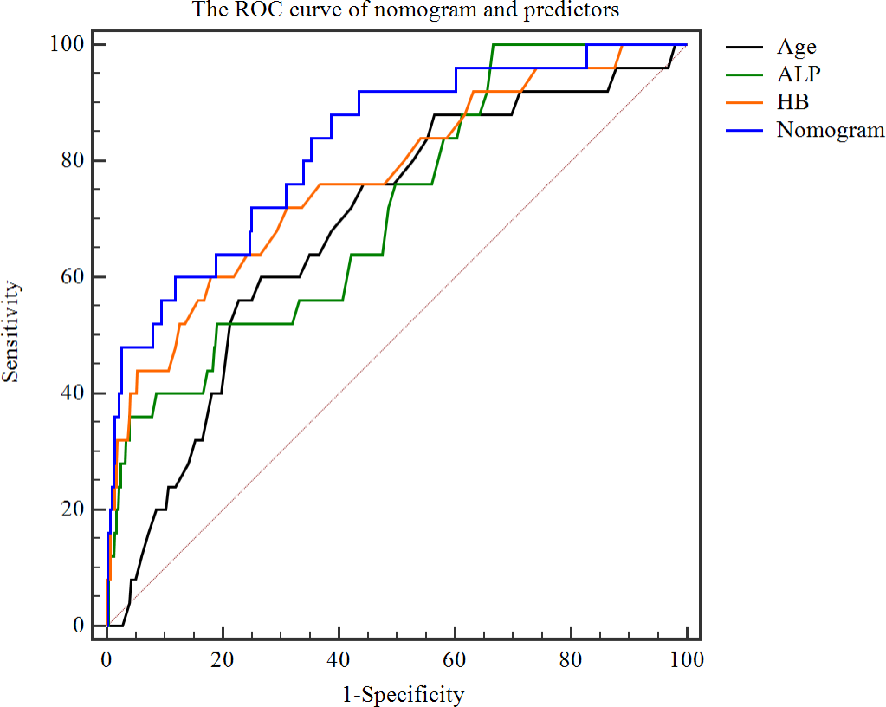
The ROC curve of nomogram and predictors.

**Figure 5:**
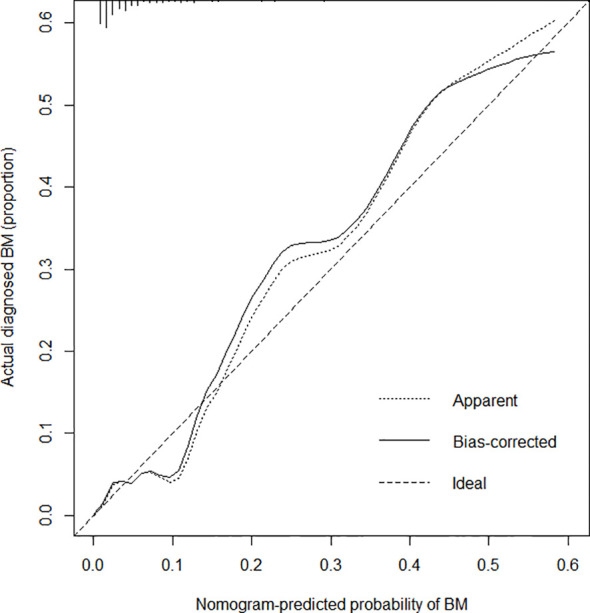
Calibration curves for the bone metastasis prediction nomogram. The x-axis represents the predicted bone metastasis risk. The y-axis represents actual diagnoses of bone metastases. The diagonal dotted line represents a perfect prediction by an ideal model. The solid line represents the performance of the nomogram, where a closer fit to the diagonal dotted line represents a better prediction.

The C-index for the prediction nomogram was 0.825, and bootstrapping validation confirmed a C-index of 0.815, indicating that the model has good discriminatory power. The ROC curve showed that nomogram had good predictive accuracy, which was higher than that of single independent predictor (**Figure 4**). The calibration curve of the nomogram for predicting of bone metastases in patients with TC demonstrated good agreement (**Figure 5**). The apparent performance of the nomogram showed good prediction capability.

### Clinical use

**Figure 6:**
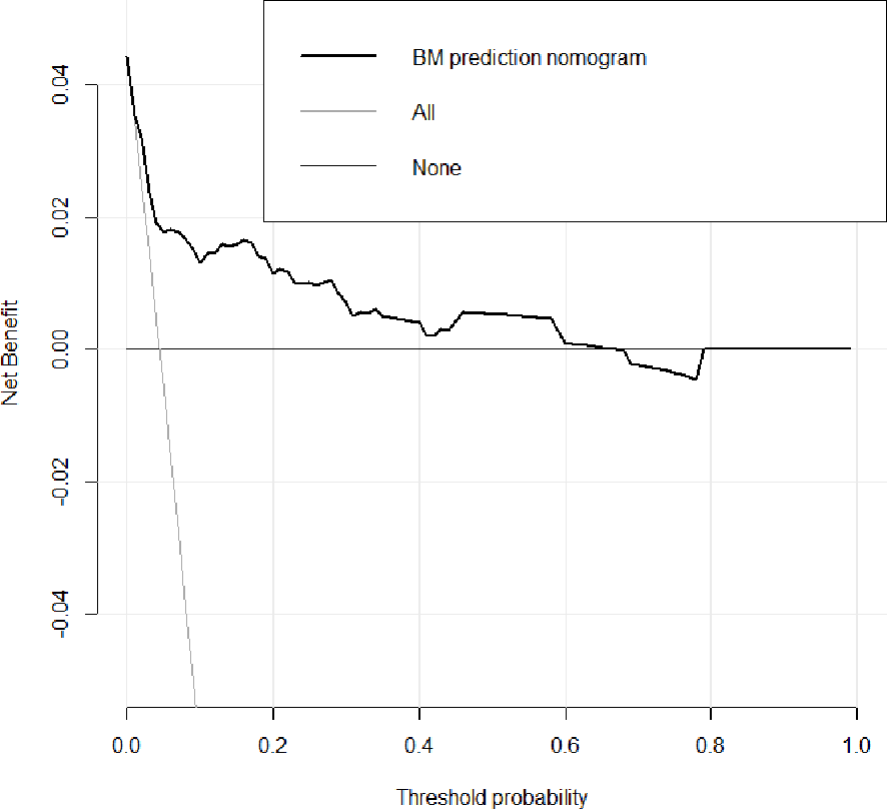
Decision curve analysis for the bone metastasis prediction nomogram. The dotted line represents the bone metastasis risk nomogram. The thin solid line represents the assumption that all patients have bone metastases. The thick solid line represents the assumption that no patients have bone metastases. The y-axis measures the net benefit. The net benefit was calculated by subtracting the proportion of all patients who are false positive from the proportion who are true positive, weighting by the relative harm of forgoing treatment compared with the negative consequences of an unnecessary treatment. Here, the relative harm was calculated by pt/(1-pt). “pt” (threshold probability) is where the expected benefit of treatment is equal to the expected benefit of avoiding treatment; at which time a patient will opt for treatment informs us of how a patient weighs the relative harms of false-positive results and false-negative results ([a - c]/[b -d] = [1 - pt]/pt); (a–c) is the harm from a false-negative result; (b–d) is the harm from a false positive result. a, b, c and d give, respectively, the value of true positive, false positive, false negative, and true negative (19). The decision curve showed that if the threshold probability of a patient or doctor is > 1% and < 67%, respectively, using the nomogram in the current study to predict bone metastases adds more benefit than the intervention-all-patients or intervention-none schemes. For example, if the personal threshold probability of a patient is 20% (ie, the patient would opt for treatment if his probability of bone metastasis was 20%), then the net benefit is 0.151 when using the nomogram to make the decision of whether to undergo treatment, with added benefit than the intervention-all-patients or intervention-none schemes. The net benefit was comparable, with several overlaps, on the basis of the nomogram.

Decision curve analysis for the nomogram is presented in **Figure 6**. It showed that if the threshold probability of a patient or doctor is > 1% and < 67%, respectively, using the nomogram developed in the current study to predict bone metastasis risk added more benefit than either the intervention-all-patients or intervention-none scheme. Within this range, the net benefit based on the nomogram was comparable, with several overlaps.

### Discussion

In previous studies, there were many studies related to bone metastasis of TC. Orita Y et al. observed bone metastases in 52 (3.7%) of 1398 patients with DTC (20). Choksi et al. studied the incidence of bone related events in thyroid cancer and found that the incidence of bone metastasis in TP was 3.9% (21, 22). Here, we found that the probability of bone metastasis in thyroid cancer was 4.21%, which was similar to previous studies. In general, the probability of bone metastasis of TC is not high, but once bone metastasis occurs, its complicated bone pain, spinal cord compression and pathological fracture will seriously affect the quality of life of patients. Bone scintigraphy is usually used to identify possible bone metastases in patients newly diagnosed with TC. However, the American Society of Clinical Oncology has reported the overuse and associated costs of BS in patients with extremely low risk of metastasis (23, 24). In recent years, many scholars have used artificial intelligence and machine learning techniques to predict cancer metastasis (25–27), and although they have better performance, they are slightly lacking in interpretability due to the black box characteristics of complex algorithms. Currently, nomograms are widely used as prognostic devices in oncology and medicine; these instruments employ user-friendly digital interfaces to increase accuracy, and provide easily understood prognoses, which facilitates better clinical decision making (17, 28). Therefore, based on the clinical data of 565 thyroid cancer patients, we identified independent risk factors for bone metastasis and constructed a nomogram model to predict the risk of BM in patients with newly diagnosed TC.

In the present study, we found three independent risk factors associated with BM, including age, ALP and HB. More importantly, based on the three variables, we developed and validated a practical dynamic nomogram for assessing the risk of bone metastases in newly diagnosed TC patients. Incorporating demographic and clinicopathological feature risk factors into an easy-to-use dynamic nomogram could facilitate prediction of bone metastases. Internal validation in the patient cohort demonstrated good discrimination and calibration of the model. The high C-index indicated that the nomogram model has high accuracy and can be widely used (28). Overall, the present study provides an accurate prediction tool for bone metastases in patients with TC.

In previous studies, age has been demonstrated to influence the prognosis of patients with TC (29–32). Further, age has been reported as a risk factor for bone metastases in patients with lung and breast cancer, and younger patients more prone to bone metastasis (33–35). However, few studies have confirmed an association between age and bone metastases in TC. In this study, we found a statistical correlation between age and bone metastases in patients with TC, and the risk of bone metastases increased with age. This may be related to genetic variation in TC cells in patients at different ages (36).

Several studies have reported that a shortage of red blood cells containing HB was a common complication in cancer patients, affecting more than 50% of cancer patients (37). Kawai et al. (38) identified HB levels is related to bone metastases associated with prostate cancer, while Henke et al. (39) reported that HB level is a significant prognostic factor in breast cancer metastases. Moreover, Chen et al. (40, 41) found the HB is an independent risk factor for bone metastases of breast and renal cell cancer. In this study, HB levels were significantly lower in patients with TC who had bone metastases than those without, which could predict the probability of bone metastasis of TC. The reason why patients with low HB levels are more prone to bone metastases may be that low HB concentrations can promote tumor cell adherence to bone marrow, thereby promoting bone metastases (38).

As a bone formation marker, total serum ALP was widely used for assessment of bone metastases in breast and prostate cancers, and can effectively reflect osteogenic activity in human patients (42, 43). Sun et al. (44) reported that bone ALP was a surrogate marker of bone metastasis in patients with gastric cancer, while Chen et al. (40) showed that ALP was a risk factor for bone metastases in breast cancer, and Huang et al. (45) reported that ALP was also a risk factor for bone metastases in bladder cancer. Rao et al. (46) showed that, tumor-derived ALP regulated epithelial plasticity, tumor growth, and disease-free survival in patients with metastatic prostate cancer. In this study, both bone-specific ALP and total ALP levels differed significantly between patients with and without bone metastases, where patients with TC and high levels of ALP were more likely to develop bone metastases.

In the analysis of risk factors, age, HB, and ALP were associated with bone metastases in patients with TC. And our nomogram suggested that advanced age, lower HB, and higher ALP may be the key individual factors that determine risk of bone metastases for patients with TC. In the model, we considered the weight of each predictor according to the statistical coefficient, and finally obtained a visual prediction chart, which could combine age, HB and ALP to help predict the probability of bone metastasis in TC patients. Further, compared with the traditional nomogram, the dynamic nomogram could easily calculate the incidence of bone metastases in patients with TC for any chosen set of values of the explanatory variables when there were higher-order interaction terms and smoother in the model. Therefore, this dynamic nomogram can be used to evaluate the risk of bone metastasis of TC simply, conveniently and quickly, and assist clinicians to make accurate clinical decisions.

However, there are some limitations in this study. Firstly it was based on retrospective data from a single institution, which will inevitably lead to inherent data bias. Thus, prospective and multi-center studies are required to validate our model. Secondly, the consistency of our nomogram was tested robustly using internal validation by bootstrap testing, but external validation is needed. Therefore, we will continue to collect cases to further validate the model, and we hope that other researchers could modify the prediction model with external data. Thirdly, clinical markers that may affect bone metastasis of cancer, including BRAF mutation, calcitonin, TERT mutation, and Ki-67 index, were not included in this study (9, 47, 48), and we will continue to expand our data collection to further improve our model in the future.

## Conclusion

In conclusion, here we identified age, ALP, and HB as independent factors of bone metastases in patients with TC. Based on this information, we developed a practical dynamic nomogram, with a relatively good accuracy, to help clinicians predict the risk of bone metastases in patients with TC. The ability to estimate of individual risk provides clinicians and patients with more power to make decisions regarding medical interventions. Nevertheless, the nomogram requires external validation, and further research is needed.

## Data availability statement

The raw data supporting the conclusions of this article will be made available by the authors, without undue reservation.

## Ethics statement

The studies involving human participants were reviewed and approved by the Ethics Committee of the First Affiliated Hospital of Nanchang University, and all participants signed written informed consent form. The patients/participants provided their written informed consent to participate in this study.

## Author contributions

W-CL conceived of and designed the study. W-CL, M-PL, W-YH, Y-XZ, and B-LS performed analysis and generated the figures and tables. W-CL and M-PL wrote the manuscript and S-HH, Z-LL, and J-ML critically reviewed the manuscript. All authors contributed to the article and approved the submitted version.
